# Overweight among children and adolescent with type I diabetes mellitus: prevalence and associated factors

**DOI:** 10.1186/s13098-016-0154-4

**Published:** 2016-07-16

**Authors:** Verônica Medeiros da Costa, Patricia de Carvalho Padilha, Géssica Castor Fontes de Lima, Aline Alves Ferreira, Jorge Luiz Luescher, Luciana Porto, Wilza Arantes Ferreira Peres

**Affiliations:** Diabetes Sector of the Instituto de Puericultura e Pediatria Martagão Gesteira (IPPMG/UFRJ), Rio de Janeiro-RJ, Brazil; Department of Nutrition and Dietetics, Instituto de Nutrição Josué de Castro (INJC/UFRJ), Rio de Janeiro-RJ, Brazil; Research Group on Maternal and Child Health (GPSMI), INJC/UFRJ, Rio de Janeiro-RJ, Brazil; Department of Social and Applied Nutrition, INJC/UFRJ, Rio de Janeiro-RJ, Brazil; Hepatology Sector of the Hospital Universitário Clementino Fraga Filho (HUCFF)/UFRJ, Rio de Janeiro-RJ, Brazil

**Keywords:** Nutritional assessment, Type 1 diabetes mellitus, Children, Adolescents, Overweight

## Abstract

**Objectives:**

Describe the overweight frequency (overweight and obesity) and identify the factors associated with this in children and adolescents with type 1 diabetes mellitus (T1DM) treated at a University Children’s Hospital in Rio de Janeiro.

**Methods:**

This is an analytical cross-sectional study, which included patients diagnosed with T1DM who had complete anthropometric data (weight and height) and excluded those using drugs with effect on weight gain, genetic syndromes, celiac disease, hypothyroidism, renal failure and other chronic diseases, and pregnant women. The data collection was referring to the last consultation, and with respect to laboratory tests, the most recent data was collected. The dependent variable was the overweight, defined as Z score ≥1. The independent variables were gender, age, insulin dose, duration of disease, lipid profile, glycated hemoglobin, type of prescribed food planning, and place of residence. A logistic regression model was built for each outcome studied, considering significant associations those with p < 0.05.

**Results:**

The study included 195 patients with a mean age of 10.6 (±3.8) years, and 49.7 % (n = 97) aged less than 10 years. The overweight frequency was 40 % (n = 78). The age ≥10 years (OR 0.41; 95 % CI 0.20–0.86; p = 0.019) and the dose of insulin/kg ideal weight (OR 3.38; 95 % CI 1:55–7:39; p = 0.002) were considered the variables associated with overweight.

**Conclusions:**

There was a high prevalence of overweight, which explains strategies for promoting healthy eating habits and changing lifestyle with a focus on children and adolescents with diabetes.

## Background

Historically, due to acute lack of insulin production, type 1 diabetes mellitus (T1DM) was associated with severe emaciation diagnosis. However, with the worldwide overweight epidemic in recent decades, there was an increase in the incidence of type 2 diabetes mellitus (T2DM), especially in adolescents, making it as common as type 1 in this age group in some countries [1, 2]. Consequently, there were also changes in the nutritional status of children and adolescents with T1DM, which may be overweight at the beginning, hindering the differential diagnosis between the two types of diabetes 1.

In a cohort study conducted in the United States, with children and adolescents with type 1 diabetes, a prevalence of 38.5 % of overweight was found, showing that the prevalence of overweight and obesity in children and adolescents with type 1 diabetes is increasing as in the general population [3]. Another American study called SEARCH found a prevalence of overweight of 34 % among young people with type 1 diabetes, not differing from non-diabetic population of the same age [4].

These data corroborate the global trend of increase of overweight in children, including the diabetic population, as evidenced by Libman et al. [5] who observed a threefold increase in the prevalence of overweight in two consecutive decades. Certainly, the presence of overweight is noteworthy since in addition to increasing cardiovascular risk, it increases insulin resistance, which can intensify complications in the short and long term, influencing the diabetes treatment [4].

In patients with type 1 diabetes, the risk factors for developing cardiovascular diseases begin in childhood and can persist into adulthood. Studies have shown the early onset of severe atherosclerosis in children with type 1 diabetes compared to healthy children due to damage caused by high blood glucose [6, 7].

It is believed that excess weight can be explained by high doses of insulin, lifestyle changes, especially those related to food intake and reducing energy expenditure, and high levels of growth hormone [8, 9]. Moreover, recent evidence highlights that overweight is not associated only with T2DM, because patients with T1DM may also have insulin resistance [9, 10].

Modern therapies with flexible schemes of insulin therapy resulted in less restrictive diet, which can represent less healthy food choices [11, 12]. Therefore, young people with T1DM should be advised about the conscious use of the method [13, 14].

Given the above, the objective of this study was to describe the frequency of overweight (overweight and obesity) and identify the factors associated with this in children and adolescents with type 1 diabetes treated at the Pediatric University Hospital of Rio de Janeiro.

## Methods

This is an analytical cross-sectional study based on retrospective data of children and adolescents diagnosed with T1DM assisted in the Diabetes Clinic of a University Children’s Hospital in the city of Rio de Janeiro. That clinic is reference center for the treatment of children and adolescents with diabetes in the city of Rio de Janeiro, consisting of a multidisciplinary team of pediatric endocrinologists, nutritionists, nurses, social workers and psychologists.

The study included all patients enrolled in the clinic database between April 2009 and December 2011, diagnosed with T1DM and complete anthropometric data (weight and height) recorded in the database, and excluded those users of drugs with effect on weight gain, genetic syndromes, celiac disease, hypothyroidism, renal failure, or other chronic diseases, pregnant women and patients with incomplete anthropometric information.

For the nutritional status diagnosis, the body mass index (BMI) was used, as recommended by the World Health Organization [15, 16] and adopted by the Ministry of Health [17]. For the statistical analysis, were created the without overweight variable (low weight and eutrophia) and the overweight variable (overweight and obesity).

The data collection was referring to the last consultation, and with respect to laboratory tests, the most recent data was collected, which according to the routine they are requested between consultations. The laboratory tests that were used in the analysis were the following: HbA1c and lipid profile (total cholesterol, low density lipoprotein (LDL), high density lipoprotein (HDL) and triglycerides). The HbA1c was measured by the high efficiency liquid chromatography method (HELC), and classified according to the recommendation by the American Diabetes Association [18] as adequate (HbAC1 < 7.5 %) and inadequate (HbAC1 ≥ 7.5 %). The lipid profile classification used was: total cholesterol (desirable if <150 mg/dL, borderline if between 150 and 169 mg/dl, increased if ≥170 mg/dL), HDL (desirable if ≥45 mg/dL, inadequate if <45 mg/dL), LDL (desirable if <100 mg/dL, borderline if between 100 and 129 mg/dl, increased if ≥130 mg/dL), and triglycerides (desirable if <100 mg/dL, borderline if between 100 and 129 mg/dL, and increased if ≥130 mg/dL) [19]. However, a variable categorized as presence (at least one lipid abnormality) or absence of dyslipidemia was created.

The dependent variable in the study was overweight (overweight—BMI between 1 and 2 Z score and Obesity BMI >2 Z score). The independent variables were: clinical characteristics (HbA1c, dyslipidemia, disease duration—more than 5 years and less than 5 years, and insulin dose per kg/ideal weight); diet (type of prescribed meal plan—traditional method of portions and carbohydrate counting method (CCHO), and sociodemographic (place of residence—Rio de Janeiro and adjacent regions, and age <10 years and ≥10 years, and gender).

Descriptive statistical procedures were performed. To examine the isolated effect of the independent variables in overweight, bivariate logistic regression was used. The covariates that showed a p ≤ 0.20 value, or the variables for potential explanation (which has shown an association with overweight in previous studies), were considered as candidates for remaining in the final model.

In the following analysis, the selection of the variables included in the final logistic model was performed by automated and manual procedures (Forward and Wald test). The interaction of the variables was verified by the correlation matrix.

The selection of the final model took into account the analysis of residues by graphical observation, deviance analysis, statistics and akaike information criteria (AIC), as well as clinical and epidemiological significance. We used the 95 % significance level for the estimated calculation of odds ratio (OR) in binary logistic regression.

The descriptive statistical analysis were performed with SPSS software for Windows version 21.0 (SPSS Inc. Chicago, USA) and the others ones in the free software R, version 3.0.1, using the epicalc library.

The study was designed respecting the expected ethical aspects. It was approved by the Research Ethics Committee of the institution under protocol number 59/11.

## Results

**Fig. 1:**
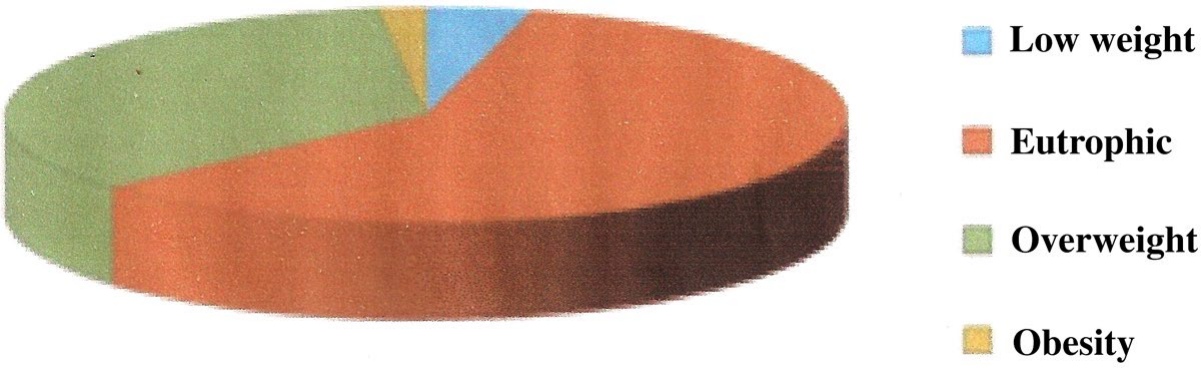
Nutritional status classification of children and adolescents with type 1 diabetes: low weight 1 % (n = 2), eutrophic 59 % (n = 115), 30.3 % overweight (n = 59) and obesity 9.7 % (n = 19)

The sample was composed of 195 children and adolescents, of whom 106 (54.4 %) males and 89 (45.6 %) females. The mean age was 10.6 (±3.8) years, 49.7 % (n = 97) aged less than 10 years. The classification of the nutritional state of the sample is shown in Fig. 1, with 1.0 % (n = 2) with low weight, 59 % (n = 115) eutrophy, 30.3 % (n = 59) with overweight, and 9.7 % (n = 19) with obesity, totaling 40 % (n = 78) with excess weight.

As for HbA1C, 181 individuals had this data, and 49.2 % (n = 89) were adequate compared to the values proposed as ideal, and the average Hb1Ac 8.07 %. The age at diagnosis, disease duration and dose of insulin/kg of ideal weight had averages of 5.54 (±2.79) years, 5.58 (±3.37) years, and 1.03 (±0.48) units, respectively. In Table 1, the average values of total cholesterol, LDL, HDL and triglycerides are represented. Regarding the lipid profile, 42.8 % (n = 71) had high total cholesterol, 15.8 % (n = 26) had inadequate HDL, 28.5 % (n = 47) and 12.5 % (n = 20) had LDL-c and elevated triglycerides, respectively, and 71.5 % (n = 125) had some change in the lipid profile.

**Table 1:** Average values of total cholesterol, LDL-C, HDL-C and triglycerides in children and adolescents with T1DM treated at a University Children’s Hospital in Rio de Janeiro

Lipid fraction	n	Minimum	Maximum	Average	SD
TC (mg/dL)	166	107	284	167.92	33.53
LDL-c(mg/dL)	165	42	197	87.04	27.98
HDL-c(mg/dL)	165	29	196	64.4	22.90
TG (mg/dL)	160	30	287	78.38	049.43

*CT* total cholesterol; *LDL*-*C* low density lipoprotein; *HDL*-*C* high density lipoprotein; *TG* triglycerides; *SD* standard deviation

The dietary planning based on CCHO was followed by 44.7 % (n = 85), and the method of portions by 55.3 % of the patients (n = 105). In the evaluation of the place of residence, it was found that the majority 68 % (n = 132) lived in the city of Rio de Janeiro.

The logistic regression models, unadjusted and adjusted for factors associated with overweight are shown in Tables 2 and 3, respectively. After adjusting the variables, the age ≥10 years was protective, and the higher dose of insulin/kg ideal weight increased the chance for the overweight. The protection related to age ≥10 years is represented by OR 0.41, which showed a relative risk lower than half for present overweight, when compared to age lower than 10 years. In contrast, higher doses of insulin increased more than three times the relative risk for overweight, when compared to the lowest dose of insulin/kg ideal weight.

**Table 2:** Prevalence and unadjusted odds ratios of the factors associated with overweight in children and adolescents in a University Children’s Hospital in Rio de Janeiro

Variables	n	OR	RI 95 %	P
*Age (years)*				
<10	42	1		
≥10	36	0.76	0.43–1.35	0.326
*Gender*				
Male	35	1		
Female	42	1.81	1.01–3.24	0.044
*Place of residence*				
Rio de Janeiro	50	1		
Surroundings	26	1.18	0.64–2.19	0.589
*Disease duration (years)*				
<5	37	1		
>5	39	0.92	0.51–1.64	0.772
*Dietary planning methods*				
Portions	40	1		
CCHO	35	1.14	0.63–2.04	0.666
*HbA1C adequacy*				
Adequate	30	1		
Inadequate	42	1.65	0.9–3.01	0.102
Daily dose of insulin/kg	–	2.12	1.13–3.97	0.019
*Changes in lipid profile*				
No	12	1		
Yes	50	1.61	0.75–3.45	0.212

**Table 3:** Odds ratio adjusted for factors associated with overweight in children and adolescents treated at the Paediatric University Hospital of Rio de Janeiro

Variables	OR	RI 95 %	P
*Age (years)*			
<10	1		
≥10	0.41	0.20–0.86	0.019
Daily dose of insuln/kg^a^	3.38	1.55–7.39	0.002

*AIC* akaike information criterion: 252.81

^a^Daily dose of insulin per kilogram of ideal body weight

## Discussion

The prevalence of overweight is high and confirms data from national and international studies. It is believed that the nutritional status of children and adolescents with T1DM reflects the results of the data found in the Brazilian population without T1DM at the same age, characterized by the representative increase of overweight with continuous reduction of the prevalence of malnutrition [20].

Luczynski et al. [21] in a sample of 500 Polish children with T1DM, found a prevalence of 30.2 % of overweight, 3.2 % of the metabolic syndrome and 4.8 % of hypertension. In Brazil, Liberatore et al. [1] and Marques et al. [8] found among children and adolescents with type 1 diabetes prevalence of overweight of 16 and 14.1 %, respectively. These studies differ due to the smaller sample size, period and criteria adopted for anthropometric assessment.

More recently, a large multicenter study in 28 public clinics in 20 Brazilian cities, identified an overweight prevalence of 31.6 % among individuals with T1DM, being mostly in the age group up to 15 years. These data are consistent with the Household Budget Survey [22], where 33.5 % of children aged 5–10 years were found to be overweight, while the prevalence among adolescents was 21.5 %. Thus, it is observed that children and adolescents with T1DM are following the epidemiological profile of overweight shown by children and adolescents without diabetes.

Noteworthy is the high frequency of dyslipidemia, consistent with findings in literature. The frequency of elevated LDL (>100 mg/dL) in other studies ranges from 20 to 50 % [4, 23]. However, the literature is poor in relation to information on the absorption and synthesis of cholesterol in T1DM. Also, it is not known the actual prevalence of dyslipidemia in children and adolescents with this disease, which hinders the study of demographics, social-economics or ethnic factors associated with it. Alves et al. reported that 32.6 % (n = 49) children and adolescents with T1DM and mean HbA1c of 11 % had some form of dyslipidemia [23].

The disease duration variable of disease was not associated with overweight. However, Jose et al. [24] found association between disease duration and insulin doses with worse control of T1DM according to the value of HbA1c. This lack of association between disease duration and overweight is justified; patients with the disease for a longer period of time and adolescents know the symptoms and can manage insulin better, so they tend to subtract insulin doses and/or meals as measures to prevent undesirable weight gain.

Age ≥10 years was protective to overweight when compared to age <10 years, possibly because at the lower age range there is a greater fear from parents regarding the episodes of hypoglycemia, which leads them to overfeed these children as prevention. On the other hand, the greater insulin dose was associated with overweight, which confirms the literature. These results justify further specific actions aimed to younger patients’ family members and overweight patients because they have higher risks of overweight and large doses of insulin, respectively, resulting in insulin resistance and increased risk of future complications.

Reinehr et al. [10], in a study including 8156 children and adolescents with type 1 diabetes on intensive insulin therapy, found a positive association between overweight and the daily dose of insulin per kilogram of ideal weight, which would explain the insulin resistance in this group with T1DM [10]. However, Liberatore et al. showed no association between the dose of insulin and BMI [1].

From a physiological point of view, insulin sensitivity must be related to lean body mass, since glucose intake occurs predominantly in muscle tissue. The diabetic patients that are overweight often require higher doses of insulin, since the glucose intake occurs about 75 % in the muscle tissue and only 4 % in the fat tissue [1, 10].

Because insulin is the key to the entry of glucose into the cell, a greater insulin demand occurs to perform this functionin the subjects with excess of fat tissue. This process relates to lower glucose intake and consequent increased demand for insulin, suggesting that the higher the fat mass, the higher the insulin resistance.

Fröhlich-Reiterer et al. [25] identified the predictor factors for BMI increase during the course of diabetes. For females, low BMI in early diabetes, intensive insulin therapy, and higher insulin dose, additionally to longer disease duration and age were associated with greater increases in BMI.

The same authors suggest that the number of injections, the amount and type of insulin, are determining factors for increased BMI. The reasons for this are probably the anabolic effect of insulin, which promotes weight gain, stimulating the lipogenesis, inhibiting protein catabolism and slowing basal metabolism, on one hand, and on the other hand, the weight gain is influenced by the caloric intake.

Children with intensive insulin therapy are likely to have a more flexible eating pattern, which could lead to increased risk of overweight. However, our study showed no association between the type of diet planning and overweight. This fact may be due to the approach taken in the CCHO prescription, which emphasizes the consumption of healthy foods, regardless of the dietary planning method. In addition, studies on nutrition in T1DM are scarce, but studies have shown that weight gain can also be influenced by diets high in fat and lower carbohydrate content to try to avoid insulin injections [26, 27].

An American study, with 35 young people between 8 and 21 years, with T1DM, showed that insulin regimens and more flexible diets are associated, according to parents and children, to frequent consumption of unhealthy foods such as high calories, low in fiber and micronutrients snacks [28]. In Brazil, an epidemiological study conducted about diet adherence, cardiovascular risk and T1DM reported that only 54.2 % of patients adhered to the diet prescribed, and those with better adherence had lower BMI, improved metabolic control (HbA1C and lipid profile), and best pressure level. The study also found lower adherence among those with high BMI [29].

We can cite as limitations of this study the absence of anthropometric measurements, such as waist circumference, measures of body composition and level of physical activity, and for not being a longitudinal study, which would allow the assessment of the evolution of anthropometric profile and its relation to the factors studied during the treatment.

## Conclusions

The increase in prevalence of risk factors associated with overweight among children and adolescents is a global health problem. As children and adolescents with type 1 diabetes have also the same risk of developing overweight, monitoring weight gain is an important aspect in the care of children and adolescents with T1DM. Especially among those of younger age and with higher doses of insulin. In this context, it is necessary to discuss strategies for promoting glycemic control in this group, especially in the group of children under 10 years, with a focus on lifestyle, which emphasizes the promotion of healthy eating habits and physical activity.

## Abbreviations

T1DM: type 1 diabetes mellitus
T2DM: type 2 diabetes mellitus
BMI: body mass index
LDL: low density lipoprotein
HDL: high density lipoprotein
HbAC1: glycated hemoglobin
HELC: high efficiency liquid chromatography
CCHO: carbohydrate counting method
AIC: akaike information criteria
